# A Randomized Controlled Trial to Compare the Effect of Peanuts and Almonds on the Cardio-Metabolic and Inflammatory Parameters in Patients with Type 2 Diabetes Mellitus

**DOI:** 10.3390/nu10111565

**Published:** 2018-10-23

**Authors:** Yun-Ying Hou, Omorogieva Ojo, Li-Li Wang, Qi Wang, Qing Jiang, Xin-Yu Shao, Xiao-Hua Wang

**Affiliations:** 1 School of Nursing, Medical College, Soochow University, Suzhou 215006, China; houyunying@suda.edu.cn (Y.-Y.H.); wanglili83476@suda.edu.cn (L.-L.W.); xuweipan@whu.edu.cn (Q.W.); 2 Faculty of Education and Health, University of Greenwich, London SE9 2UG, UK; o.ojo@greenwich.ac.uk; 3 Medical College, Soochow University, Suzhou 215006, China; jiangqing2015@suda.edu.cn (Q.J.); wudan@suda.edu.cn (X.-Y.S.)

**Keywords:** type 2 diabetes mellitus, peanut, almond, glycemic control, body mass index, lipids, interleukin-6

## Abstract

A low carbohydrate diet (LCD), with some staple food being replaced with nuts, has been shown to reduce weight, improve blood glucose, and regulate blood lipid in patients with type 2 diabetes mellitus (T2DM). These nuts include tree nuts and ground nuts. Tree nut consumption is associated with improved cardio-vascular and inflammatory parameters. However, the consumption of tree nuts is difficult to promote in patients with diabetes because of their high cost. As the main ground nut, peanuts contain a large number of beneficial nutrients, are widely planted, and are affordable for most patients. However, whether peanuts and tree nuts in combination with LCD have similar benefits in patients with T2DM remains unknown; although almonds are the most consumed and studied tree nut. This study sought to compare the effect of peanuts and almonds, incorporated into a LCD, on cardio-metabolic and inflammatory measures in patients with T2DM. Of the 32 T2DM patients that were recruited, 17 were randomly allocated to the Peanut group (*n* = 17) and 15 to the Almond group (*n* = 15) in a parallel design. The patients consumed a LCD with part of the starchy staple food being replaced with peanuts (Peanut group) or almonds (Almond group). The follow-up duration was three months. The indicators for glycemic control, other cardio-metabolic, and inflammatory parameters were collected and compared between the two groups. Twenty-five patients completed the study. There were no significant differences in the self-reported dietary compliance between the two groups. Compared with the baseline, the fasting blood glucose (FBG) and postprandial 2-h blood glucose (PPG) decreased in both the Peanut and Almond groups (*p* < 0.05). After the intervention, no statistically significant differences were found between the Peanut group and the Almond group with respect to the FBG and PPG levels. A decrease in the glycated hemoglobin A1c (HbA1c) level from the baseline in the Almond group was found (*p* < 0.05). However, no significant difference was found between the two groups with respect to the HbA1c level at the third month. The peanut and almond consumption did not increase the body mass index (BMI) and had no effect on the blood lipid profile or interleukin-6 (IL-6).In conclusion, incorporated into a LCD, almonds and peanuts have a similar effect on improving fasting and postprandial blood glucose among patients with T2DM. However, more studies are required to fully establish the effect of almond on the improvement of HbA1c.

## 1. Introduction

Type 2 diabetes mellitus (T2DM) is a chronic disease that involves a heterogeneous group of disorders of the intermediary metabolism, characterized by glucose intolerance [1]. The incidence and prevalence of T2DM have increased markedly worldwide, and its complications are the leading causes of morbidity and premature mortality [2]. The use of diet in the prevention, treatment, and control of T2DM has been recommended, and is one of the strategies for managing the condition. According to the American Diabetes Association (ADA), the nutritional goals for patients with T2DM are to achieve normoglycemia and a cardio protective lipid profile that reduces the risk for cardiovascular disease (CVD) [3].

In recent years, a low carbohydrate diet (LCD) has gained popularity [4],and its effectiveness in reducing weight, improving blood glucose, and regulating blood lipid in patients with T2DM has been confirmed by the American Diabetes Association and Diabetes UK [5,6]. ALCD combined with a low saturated fat intake may be best for patients [7].

Nuts are high in unsaturated fat and are a rich source of bioactive nutrients that have the potential to provide metabolic and cardiovascular benefits [8]. Bodies concerned with diabetes and CVD (e.g., the Canadian Cardiovascular Society and the European Atherosclerosis Society) are now advocating for an increase in nut consumption as part of their dietary recommendations [9,10,11,12].

Nuts include tree nuts (almond, walnut, hazelnut, pistachio, pine nut, cashew, pecan, macadamia, and Brazil nut) and ground nuts (mainly peanut). Almonds are the most studied tree nut. Clinical trials have shown that the consumption of almonds as well as other tree nuts is associated with improved glycemic control, insulin sensitivity, decreased inflammation, and reduced/sustained body weight [13,14,15,16]. However, tree nuts are difficult to promote in patients with diabetes because of their high cost, especially in developing and under-developed countries.

As the main ground nut, peanuts have a similar nutrient composition to tree nuts, thus being nutrient-dense and rich in monounsaturated fatty acid (MUFA) (40% of energy).They are also a good source of arginine, fiber, phytosterols, polyphenols, niacin, folic acid, and vitamin E [17]. In addition, peanuts are widely planted and are much cheaper than tree nuts, and they are affordable for most T2DM patients. Randomized controlled and cross-over trials have shown that peanut consumption helps to moderate glucose concentrations [18], improve the postprandial lipid response, and preserve endothelial function [19]. However, whether peanuts and tree nuts have similar benefits forT2DM patients remains unknown.

Almonds are the most consumed tree nut [20]. We aim to compare the effect of peanuts and almonds incorporated into LCD on cardio-metabolic and inflammatory measures in T2DM patients.

## 2. Materials and Methods

### 2.1. Subjects

The participants were recruited from a diabetes club and from the Endocrine Division of the First Affiliated Hospital of Soochow University. The inclusion criteria were as follows: The patients were diagnosed with T2DM, had glycated hemoglobin A1c (HbA1c) of less than 10%, had no change in oral antidiabetic drugs or in insulin half a month before the intervention, were between 40 to 80 years old [15,21], were able to communicate, had volunteered to participate in this study, and were willing to provide informed consent. Those that were excluded were the patients who ate nuts regularly (≥four per day/week) [22]; were allergic to nuts or other food; had difficulty in chewing nuts as a result of fewer teeth or for other reasons; could not adhere to a LCD strategy; received other dietary interventions; had severe conditions including indigestion, hepatic failure, renal failure, severe gallbladder and pancreatic diseases, stroke, malignant tumors, or unstable cardiovascular diseases (such as myocardial infarction, ketosis, or hyperthyroidism); those who were taking glucocorticoid; and those whose fasting blood glucose (FBG) was more than 16.7 mmol/L [23] during the intervention.

### 2.2. Study Design

This study is a prospective, randomized controlled trial (RCT) that was performed between December 2015 and April 2016. The recruited patients were randomly allocated to the Peanut and Almond groups using a table of random numbers. The random numbers were generated by one researcher, and were concealed to the researcher who was responsible for the allocation, and the participants were blinded prior to assignment. Before the intervention, all of the subjects underwent a one-week washout period [23] to diminish the effect of background diets on the study. This study followed the Declaration of Helsinki and the Guidelines for Good Clinical Practice, and was approved by the ethics committee of the First Affiliated Hospital of Soochow University (no. 2015106). All of the enrolled participants signed a consent form.

### 2.3. Sample Size Calculation

Evidence from the literature showed that the mean difference of the changes in the HbA1c levels were 1.6% between the Peanut group and the Almond group [21,24]. Therefore, we calculated 13 participants for each group, with α = 0.05 and power = 0.80. In light of the sample loss of 10%, the number for each group was calculated to be 16. Finally, we recruited 15 participants for the Peanut group and 17 participants for the Almond group in this study.

### 2.4. Intervention

We incorporated peanuts or almonds into a low-carbohydrate diet (LCD), which is a dietary strategy that refers to a carbohydrate intake of between 30–200 g/day or calories from carbohydrates/total calories <45%, being supplemented instead with fat or protein [24]. Our team developed a LCD education handbook [24] for patients with T2DM based on evidence from the literature, guidelines regarding T2DM dietary management, consultation with diabetes experts and nutritionists, and reviews by patients. The researcher and patients reviewed the LCD handbook, and the researcher trained the patients to restrict their intake of starchy staple food (such as potatoes and broad beans) to 50 g/meal per day during the one-on-one education session. The reduced staple food/meal was substituted by consuming 60 g/day peanuts for men and 50 g/day for women in the Peanut group [25], and 55 g/day almonds for men and 45 g/day for women in the Almond group [25,26]. The peanuts and almonds (without salt and with the skin intact, and free of charge) were prepared in vacuum packing, according to a daily amount. The patients were instructed to consume nuts between meals or with breakfast, or when hungry. For those whose fasting plasma glucose were higher than normal (>6.1 mmol/L), the nuts were required to be consumed before 10:00 a.m. in the morning [27]. The patients were instructed to consume 50% of the nuts before bedtime if there was a risk of a nocturnal hypoglycemic event. The intervention duration was three months for the two groups.

The follow-ups were conducted once a week in the first month of the intervention, and once every two weeks in the second and third months. The duration of each follow-up session was about 10 min. The patients’ compliance to the dietary plan was reviewed and those with a poor compliance were supported in order to adhere to the plan. The data relating to modification of hypoglycemic agents and the occurrence of hypoglycemia were also collected. Those whose diets did not meet the requirements of the dietary program in the intervention period were excluded from the study.

### 2.5. Diet Record

The patients maintained a diet record, including details of the diet of any day over the weekend and two working days, as well as the time of nut consumption. The types and quantities of the food consumed were assessed to determine the patients’ adherence to a LCD strategy. Among the patients who met the dietary requirements for a LCD, the number of bags of nuts consumed per week was assessed to determine the patients’ dietary adherence.

### 2.6. Cardio-Metabolic and Anthropometric Parameters

The cardio-metabolic and anthropometric parameters included the FBG, postprandial 2-h blood glucose (PPG) levels, HbA1c, total cholesterol, low density lipoprotein cholesterol (LDL-C), high density lipoprotein cholesterol (HDL-C), triglycerides, and body mass index (BMI). The HbA1c, blood lipids, and BMI were measured at the baseline and at the end of the third month. Venous blood samples were collected at the School of Nursing of Soochow University after a 12 h overnight fast. The HbA1c was measured by high-performance liquid chromatography using Afinion AS100 Analyzer (Alere, Inc., Shanghai, China), and the total cholesterol, LDL-C, HDL-C, and triglycerides were measured using the spectrophotometry method in the molecular laboratory of the School of Nursing of Soochow University. The height and weight were measured using a calibrated stadiometer, and the patients were weighed wearing light clothing and without shoes. The BMI was calculated as the weight (in kilograms) divided by the square of the height (in meters). The FBG and PPG levels were measured by collecting the peripheral blood from the fingers using a rapid glycemic apparatus by patients once a week at home. The glycemic meters were checked by the research staff and the patients were educated to measure the FBG and PPG correctly so as to reduce the subject bias.

### 2.7. Hypoglycemic Episodes and Antidiabetic Medication Modification

Hypoglycemic episodes in this study were determined by the patients’ self-reported hypoglycemic symptoms, with or without a measured plasma glucose concentration of ≤70 mg/dL (≤3.9 mmol/L), according to the definition of hypoglycemia in diabetes, given by the American Diabetes Association [28]. To assess the modification of the hypoglycemic agents, the use and changes of the doses of oral antidiabetic drugs and insulin were recorded at the baseline and in the third month.

### 2.8. Interleukin-6

Interleukin-6 (IL-6) was measured to assess the impact of peanuts and almonds on inflammation. The fasting venous blood was collected and the serum was separated in the molecular laboratory of the School of Nursing of Soochow University. Human IL-6 ELISA kit (R&D Systems^TM^, Emeryville, CA, USA) was used to determine the IL-6 levels in the Hematology Center of the Cyrus Tang Medical Institute at Soochow University.

### 2.9. Ratio of Urinary Albumin/Creatinine

In order to determine the safety of peanuts and almonds in patients with diabetes, a mid-stream specimen of random urine was collected, and the ratio of urinary albumin/creatinine (ACR) was measured using a dry immune marker scattering quantitative method [29].

### 2.10. Statistical Analysis

A statistical analysis was performed using SPSS 18.0 software (SPSS, Inc., Dhicago, IL, USA). For continuous variables, the results were presented as mean ± standard deviation (SD). Comparisons were performed using a *t*-test for the independent samples for general baseline demographic, and clinical characteristics, and one-way analysis of variance (ANOVA) for the outcomes of interest. To eliminate the problem of imbalance in the baseline characteristics, the comparisons of anthropometric and metabolic variables between the groups after the intervention were performed using a covariance analysis with the baseline measurements adjusted. The trends in the dietary adherence, FBG, and PPG in the two groups, which were assessed once a week during the intervention, were analyzed using repeated ANOVA, and have been presented as a fold line diagram. The intention-to-treat (ITT) of HbA1c was performed so as to ensure the reliability of the research results. For the categorical variables, the results were presented as numbers and percentages, and comparisons between the groups were performed using the Chi-squared test or the Fisher’s exact test. A *p* value of <0.05 was considered statistically significant.

## 3. Results

### 3.1. Study Participants

**Figure 1:**
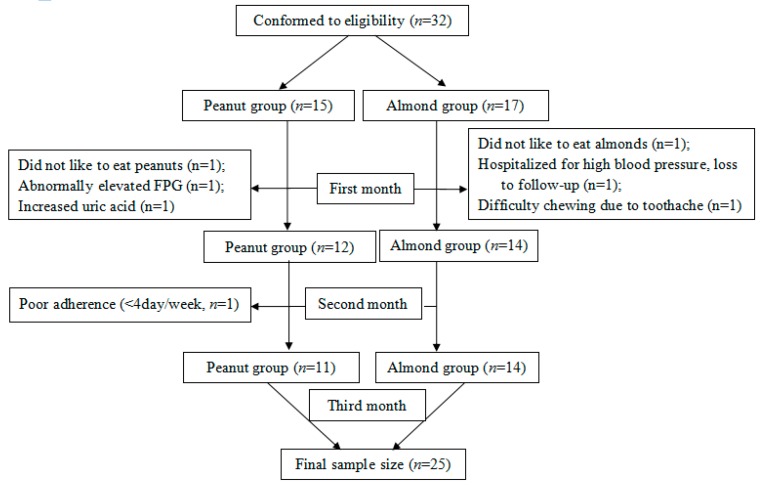
Flow diagram of the patients included in the study.

On the basis of inclusion and exclusion criteria, 32 T2DM participants were recruited and randomly allocated to the Peanut group (*n* = 15) and the Almond group (*n* = 17). Four participants in the Peanut group and three participants in the Almond group withdrew from the study. In the Peanut group, one participant did not like peanuts, one showed abnormally elevated FPG after the first week, one’s uric acid increased during the second week (with a history of increased uric acid), and one showed a poor adherence (<four per day/week). In the Almond group, one did not like almonds, one was lost to follow-up, and one could not adhere to the diet program because of toothache. Finally, the data of 11 patients in the Peanut group and 14 in the Almond group were analyzed (Figure 1). The mean age of the participants was 69.60 ± 7.25 years, and 15 (60.0%) were men. The general characteristics of the enrolled participants in each group are shown in Table 1. There were no statistically significant differences in any of the parameters between the two groups (*p* > 0.05, Table 1). The time of exercise per week in the two groups did not change significantly from the baseline, and there was no statistically significant difference found at the third month.

### 3.2. Dietary Adherence

**Figure 2:**
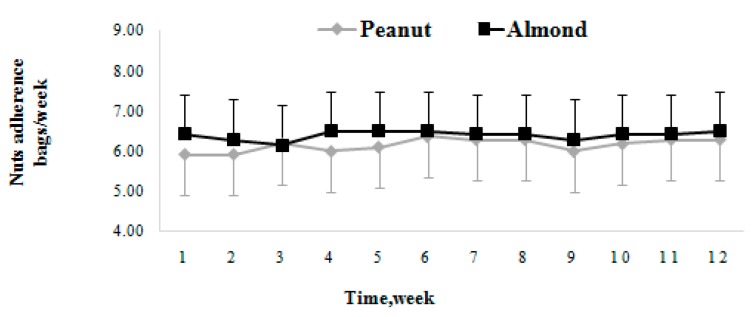
The changing trends of dietary adherence in the Peanut and Almond groups. Values are means, with their standard deviations represented by vertical bars.

The dietary adherence was assessed through the bags of nuts consumed by the participants per week. A fold line diagram was performed to compare the dietary compliance between the two groups (Peanut versus Almond). The results showed that there were no significant differences in the self-reported dietary compliance per week between the two groups (*p* > 0.05, Figure 2).

### 3.3. Effect of Peanuts and Almonds on Glycemic Control

#### 3.3.1. Fasting Blood Glucose

##### Changing Trends of Fasting Blood Glucose

**Figure 3:**
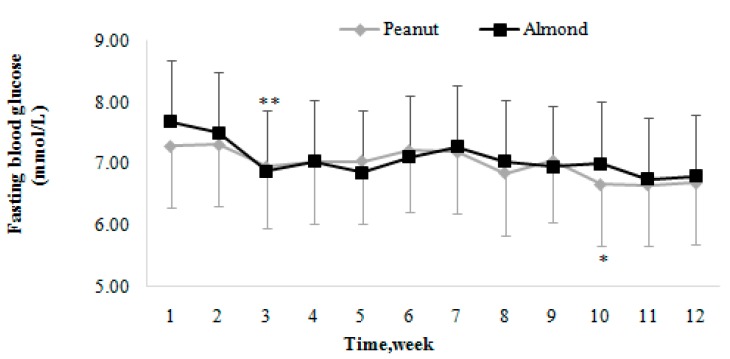
The changing trends of fasting blood glucose (FBG) in the Peanut and Almond groups. Values are means, with their standard deviations represented by vertical bars. For the Peanut group, * FBG was significantly lower at the tenth week than that at the sixth week (*p* = 0.035). For the Almond group, ** FBG was significantly lower at the third week than that at the first week (*p* = 0.001).

The changing trends of FBG in the two groups during the intervention are described by the fold line diagram (Figure 3). The results show that, for the Peanut group, the levels of FBG were stable with a slight decline, down to the tenth week to the lowest level. For the Almond group, the levels of FBG decreased significantly for the first three weeks, and then fluctuated around the level of the third month.

##### Comparison of Fasting Blood Glucose Levels

Compared to the baseline, the FBG levels of the two groups decreased significantly (*p* < 0.05). However, the differences between the two groups, with respect to FBG, were not statistically significant (*p* > 0.05) (Table 2).

#### 3.3.2. Postprandial Two-Hour Blood Glucose

##### Trends in Postprandial Two-Hour Blood Glucose

**Figure 4:**
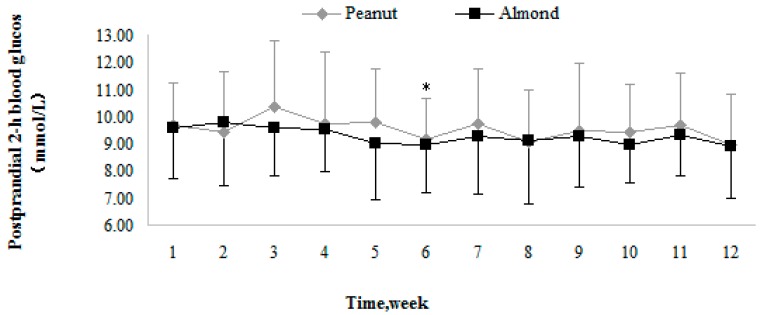
The changing trends of postprandial 2-h blood glucose in the Peanut and Almond groups. Values are means, with their standard deviations represented by vertical bars. For the Peanut group, * PPG was significantly lower at the sixth week than that at the third week (*p* = 0.027).

The changing trends of PPG in the two groups during the intervention are described by the fold line diagram (Figure 4). Both of the groups showed fluctuation, and the amplitude of the fluctuation of the Peanut group was significantly larger than that of the Almond group.

##### Comparison of Postprandial Two-Hour Blood Glucose

Compared to the baseline, the PPG in the two groups improved significantly (*p* < 0.05). However, there were no significant differences between the two groups at the third month (*p* > 0.05) (Table 3).

#### 3.3.3. Glycated Hemoglobin

At the baseline, the HbA1c levels were not significantly different between the Peanut and Almond groups. Compared with the baseline, the HbA1c decreased significantly in the Almond group (*p*< 0.05, Table 4). However, there were no significant differences between the two groups by the third month. The intention-to-treat (ITT) in relation to the HbA1c levels were performed so as to ensure the stability of the above results. The ITT results were found to be in agreement with the earlier findings (Table 5).

### 3.4. Effect of Peanuts and Almonds on Other Cardio-Metabolic and Anthropometric Indicators

Compared with the baseline, the BMI, total cholesterol, LDL-C, HDL-C, and triglycerides in the two groups did not improve significantly by the third month. After the intervention, the cardio-metabolic and anthropometric indicators were not significantly different between the two groups (Table 6).

### 3.5. Hypoglycemia and Medication Changes

#### 3.5.1. Incidence of Hypoglycemia

The incidence of hypoglycemia in the two groups showed no significant differences during the three months before the intervention (baseline) and during the intervention period. One patient in the Almond group and none in the Peanut group sustained a hypoglycemic episode during the trial.

#### 3.5.2. Antidiabetic Drugs Used

During the study, one subject in the Peanut group and two subjects in the Almond group had a decrease in the dose of oral hypoglycemic drugs, and one subject in the Almond group had an increase in the dose of oral hypoglycemic drugs, according to the recommendations of physicians. There were no significant differences in the antidiabetic drugs used between the two groups at baseline and by the third month (Table 7).

### 3.6. Effect of Peanuts and Almonds onInterleukins-6

Compared with the baseline, theIL-6 in the two groups did not improve significantly by the third month. After the intervention, the IL-6 was not significantly different between the two groups (Table 8).

### 3.7. Effect of Peanuts and Almonds on Ratio of Urinary Albumin/Creatinine

There were no significant differences between the two groups at baseline and by the third month (Table 9).

## 4. Discussion

This is the first study that compared the effect of peanuts and almonds in patients with T2DM, when incorporated into a LCD diet in order to replace some staple food. The diet diaries revealed that the participants had a good adherence to the dietary intervention, and no significant difference with respect to nut adherence was found between the two groups. This RCT showed that, in combination with a LCD diet, peanuts yielded similar reductions in FBG and PPG compared to almonds.

### 4.1. Effect of Peanuts and Almonds on Glycemic Control

High levels of FBG, PPG, and HbA1c are some of the most difficult challenges faced by patients with T2DM, and these parameters could be used as the main indicators in order to establish a glycemic control [30]. This study showed that both peanuts and almonds incorporated into a LCD diet resulted in reductions in FBG and PPG after the intervention, which is consistent with previous research results [18,21,31]. The reason might lie in the fact that there is a decrease in the total amount of carbohydrate rich foods in a LCD. In addition, peanuts and almonds are rich in fat, they possess a low-glycemic index, and could alter the glycemic index of co-consumed foods [32]. What is more, the greater fat availability may reduce the gastric emptying rate, and may decrease the carbohydrate absorption rate [33].

However, the content of unsaturated fatty acids (UFAs) and soluble fiber in almonds is higher than that in peanuts [17]. UFAs could facilitate the movement of the glucose receptor to the cell surface, thus increasing the insulin sensitivity [34]. UFAs also act through the stimulation of GLP-1 secretion, which improves the efficacy of the β-cell function [35]. Soluble fiber increases the gastric distention, viscosity in the gastrointestinal tract, and the slower absorption of macronutrients [36]. In this way, it lowers the speed of carbohydrate absorption and the concentration of PPG [37]. Based on the above reasons, the glycemic effect of almonds may be more stable than that of peanuts. In our study, although peanuts and almonds yielded similar reductions in FBG and PPG by the end of the three-months intervention, the amplitude of the fluctuation of the PPG in the Peanut group was significantly larger than that of the Almond group.

The HbA1c level can reflect the mean blood glucose level over the last 8–12 weeks, and can be used to evaluate the long-term glycemic control of patients [30]. HbA1c has a closer association with PPG than FPG [38]. In the present study, the effect of peanuts on the HbA1c reduction was not significant. This might be due to the fluctuation of PPG in the Peanut group. The result of our study is in line with the RCT by Wien et al. [39], which did not find that incorporating peanuts into an American Diabetes Association meal plan had a significant effect in decreasing HbA1c in adults with T2DM. Although there was a 0.48% decrease in HbA1c from the baseline caused by almond consumption, the greater effect of almonds on the improvement of HbA1c was not found by the third month, compared to peanuts. The short-term duration of the follow-up may be one of the reasons for this. After a 24-week almond intervention, Gulati et al. [40] found a statistically significant improvement in the levels of HbA1c compared with the control diet.

### 4.2. Other Cardio-Metabolic Indicators

The consumption of peanuts and almonds has not been associated with increased body weight, despite their high lipid content. Human feeding trials have shown that nut ingestion moderates appetite postprandially [41]. The inclusion of peanuts and almonds increases a feeling of satiety and leads to a strong dietary compensation effect. In addition, because of the inefficiency in energy absorption, nut consumption does not promote a greater energy intake than other foods [41]. In a randomized cross-over study, after 12 weeks of incorporating high oleic peanuts into the diet, a less than predicted increase in the body weight was found, despite a large additional amount of energy being consumed from the peanuts [25]. Similarly, Li et al. [26] and Gulati et al. [40] also reported no changes in the body weight and BMI with the almond diet, however, a statistically significant improvement was seen in the body fat [26], waist circumference, and waist-to-height ratio [40]. Sato et al. [42] reported a significant improvement of BMI on a LCD diet on in T2DM patients with higher baseline levels of BMI (26.5 Kg/m^2^). Among the subjects with a relative normal baseline BMI, this study did not find that peanuts or almonds incorporated into a LCD diet had a significant reduction on the BMI.

The total cholesterol, LDL-C, HDL-C, and triglyceride levels were not altered significantly with an almond or peanut diet in this study, contrary to many other studies [14]. However, the cholesterol lowering effects of nuts are shown to be the greatest in individuals with higher baseline lipids [43]. The subjects in this study had an average healthy baseline lipid level. Barbour et al. [25] and Wien et al. [39] also reported no differences in lipids with peanut consumption in subjects with healthy baseline lipid levels.

### 4.3. Hypoglycemia and Medication Changes

The antidiabetic drugs and insulin doses used in the Peanut and Almond groups were identical. There was no interference caused by the agents when comparing the glycemic control effects between peanuts and almonds in this study.

We used hypoglycemia as a safety indicator. Although there was no significant difference in the between-group comparison, the percentage of hypoglycemia was reduced from 18.2% to zero in the Peanut group. Nocturnal hypoglycemia occurred in two participants in the Peanut group before the intervention, and it was recommended that they consume 50% of their prescribed peanuts before bedtime. The occurrence of hypoglycemia in the morning causes the body to produce a large amount of glucocorticoids, namely the Somogyi effect, leading to increased blood glucose in the morning [44]. Peanuts and almonds, which are rich in healthy fat, can delay the speed of gastric emptying, and can continuously supply energy [27] to the body so as to prevent the occurrence of the Somogyi effect. During the intervention in the Almond group, daytime hypoglycemia occurred in one case because of strenuous exercise, and there was no nocturnal hypoglycemia found.

### 4.4. Effect of Peanuts and Almonds on Interleukin-6

IL-6 was chosen as an indicator of inflammation. Chronic low-level inflammation plays an important role in the occurrence and development of DM [17,45]. IL-6 is the source of the metabolic syndrome induced by inflammation, and plays a core regulatory role in the inflammatory response [46,47]. IL-6 can inhibit insulin signaling transduction and could therefore impede the action of insulin [47].

Peanuts are rich in folic acid, which can inhibit the cascade of a reaction in the process of inflammation in vessel wall, and this may reduce the release of vascular inflammatory factors [48]. Although the administration of folic acid can cause a decrease in the concentration of homocysteine, and, as a consequence, could influence the decrease in the concentration of the indicators of inflammation [49], a decrease of IL-6 caused by peanut consumption was not found in this study. As an indicator of inflammation, the C reactive protein (CRP) did not improve after 12-week of peanut consumption in the study by Barbour et al. [25]. Contrary to our study, in the study by Gulati et al. [40], a significant improvement in the CRP was found after 24-weeks of almond consumption. Whether the inconsistency in the improvement of inflammation between studies is correlated to the different intervention duration needs to be further verified.

### 4.5. Effect of Peanuts and Almonds on Kidney Burden

ACR is a sensitive indicator of early renal damage [50], which is used to assess the impact of peanuts and almonds incorporated into a LCD on renal function in this study. Our study found that peanut and almond consumption with a LCD did not increase the burden on the kidney. Díaz-López et al. also reported no change in ACR after a one-year Mediterranean diet supplemented with nuts [51].

## 5. Limitation

There are some limitations to this study. Firstly, the sample size was small. There was also an imbalance in the gender, diabetes duration, and baseline HbA1c level between groups, although there were no significant differences found. The prolonged effect of peanuts and almonds on the prognosis of T2DM was not observed because of the short follow-up duration. Finally, measurement differences might exist in the FBG and PPG levels, which were measured by the patients themselves, using different blood glucose meters at home.

## 6. Conclusions

Incorporated into a low-carbohydrate diet, both peanuts and almonds can improve the fasting blood glucose and postprandial 2-h blood glucose in patients with T2DM. The effect of almonds in promoting long-term glycemic control needs to be confirmed by more studies.

## Figures and Tables

**Table 1:** Baseline characteristics.

Variables		Peanut (*n* = 11)	Almond (*n* = 14)	*t*/*χ*^2^	*p*
		$\bar{x}$ ± SD/*n* (%)	$\bar{x}$ ± SD/*n* (%)		
**Demographic data**					
Age (years)		68.00 ± 5.80	70.86 ± 8.21	−0.977 ^a^	NS
Gender—male		5 (45.5)	10 (71.4)	- ^b^	NS
Exercise (min/week)		430.9 ± 222.2	421.4 ± 318.5	0.084 ^a^	NS
Exercise habits	Never regular exercise	4 (36.4)	10 (71.4)	- ^b^	NS
	Regular exercise	7 (63.6)	4 (28.6)		
Like sweets or rice or noodles—no		1 (9.1)	1 (7.1)	- ^b^	NS
The amount of staple food, liang/day (1 liang = 50 g)		3.77 ± 1.75	3.61 ± 2.02	0.215 ^a^	NS
Consuming nuts—yes		9 (81.8)	13 (92.9)	- ^b^	NS
**Clinical data**					
Smoking—yes		2 (18.2)	0 (0)	- ^b^	NS
SBP (mmHg)		130.73 ± 7.56	128.00 ± 13.77	0.589 ^a^	NS
DBP (mmHg)		79.55 ± 10.25	75.71 ± 8.89	1.000 ^a^	NS
Family history of diabetes—yes		5 (45.5)	6 (42.9)	- ^b^	NS
Diabetes duration, years		11.27 ± 6.36	15.21 ± 8.82	−1.247 ^a^	NS
Complications—yes		4 (36.4)	6 (42.9)	- ^b^	NS
Accompanying diseases—yes		9 (81.8)	7 (50.0)	- ^b^	NS

*p*-value for comparisons between the treatment diets by an independent samples *t*-test or Chi-square test. ^a^*t*-test; ^b^ Fisher’s exact test. SBP—systolic blood pressure; DBP—diastolic blood pressure; NS: Differences were not significant. SD—standard deviation. Complications included diabetic retinopathy, nephropathy, neuropathy, cardiopathy, foot ulcers, and cognitive impairment.

**Table 2:** Comparison of fasting blood glucose (mmol/L) between the two groups.

Study Period	Peanut (*n* = 11)	Almond (*n* = 14)	*F*	*p*
Baseline	7.73 ± 1.19	8.28 ± 2.05	0.537	NS
Third month	6.69 ± 0.54 (adjusted: 6.77 ± 0.20)	6.79 ± 0.92 (adjusted: 6.73 ± 0.17)	0.016	NS
*F*	6.945	5.785	-	-
*p*	0.016 *	0.024 *	-	-

*F*-value and *p*-value for comparisons by one-way analysis of variance or covariance analysis for between-group differences at the third month, with adjusted data presented as mean ± standard error. * *p *< 0.05; NS: differences were not significant.

**Table 3:** Comparison of postprandial 2-h blood glucose (mmol/L) in the two groups.

Study Period	Peanut (*n* = 11)	Almond (*n* = 14)	*F*	*p*
Baseline	10.36 ± 1.40	10.61 ± 2.83	0.072	NS
Third month	8.94 ± 1.55 (adjusted: 9.03 ± 0.38)	8.91 ± 1.89 (adjusted: 8.85 ± 0.34)	0.115	NS
*F*	5.011	4.487	-	-
*p*	0.037 *	0.044 *	-	-

*F*-value and *p*-value for comparisons by one-way analysis of variance or covariance analysis for between-group differences at the third month, with adjusted data presented as mean ± standard error. * *p* < 0.05; NS: differences were not significant.

**Table 4:** Comparison of glycated hemoglobin (%) between the two groups.

Study Period	Peanut (*n* = 11)	Almond (*n* = 14)	*F*	*p*
Baseline	6.81 ± 0.82	7.39 ± 1.16	0.072	NS
Third month	6.76± 0.91 (adjusted: 6.97 ± 0.15)	6.81 ± 0.73 (adjusted: 6.65 ± 0.13)	2.453	NS
*F*	0.015	4.541	-	-
*p*	NS	0.043 *	-	-

*F*-value and *p*-value for comparisons by one-way analysis of variance or covariance analysis for between-group differences at the third month, with adjusted data presented as mean ± standard error. * *p* < 0.05; NS: differences were not significant.

**Table 5:** Glycated hemoglobin (%) between the two groups in intention-to-treat (ITT).

Study Period	Peanut (*n* = 15)	Almond (*n* = 17)	*F*	*p*
Baseline	6.96 ± 0.89	7.36 ± 1.07	2.119	NS
Third month	6.93 ± 0.96 (adjusted: 6.90 ± 0.18)	6.88 ± 0.71 (adjusted: 6.65 ± 0.11)	2.361	NS
*F*	0.015	4.210	-	-
*p*	NS	0.048 *	-	-

*F*-value and *p*-value for comparisons by one-way analysis of variance or covariance analysis for between-group difference at the third month, with adjusted data presented as mean ± standard error. * *p* < 0.05; ITT—intention-to-treat; NS: differences were not significant.

**Table 6:** Comparison of other cardio-metabolic indicators between the two groups.

Variables	Study Period	Peanut (*n* = 11)	Almond (*n* = 14)	*F*	*p*
BMI (Kg/m^2^)	Baseline	22.84 ± 2.48	24.08 ± 3.15	1.141	NS
	Third month	22.67 ± 2.44 (adjusted: 23.30 ± 0.22)	23.43 ± 2.90 (adjusted: 22.94 ± 0.20)	1.482	NS
	*F*	0.025	1.141	-	-
	*p*	NS	NS	-	-
Total cholesterol (mmol/L)	Baseline	4.48 ± 0.77	4.90 ± 1.00	1.362	NS
	Third month	4.25 ± 0.93 (adjusted: 4.40 ± 0.21)	4.51 ± 0.86 (adjusted:4.39 ± 0.19)	0.002	NS
	*F*	0.398	1.260	-	-
	*p*	NS	NS	-	-
LDL-C (mmol/L)	Baseline	2.48 ± 0.72	2.97 ± 0.84	2.290	NS
	Third month	2.51 ± 0.84 (adjusted: 2.69 ± 0.15)	2.74 ± 0.63 (adjusted: 2.59 ± 0.14)	0.234	NS
	*F*	0.006	0.653	-	-
	*p*	NS	NS	-	-
HDL-C (mmol/L)	Baseline	1.49 ± 0.28	1.36 ± 0.30	1.219	NS
	Third month	1.53 ± 0.22 (adjusted: 1.49 ± 0.05)	1.34 ± 0.26 (adjusted: 1.38 ± 0.04)	3.123	NS
	*F*	0.197	0.029	-	-
	*p*	NS	NS	-	-
Triglycerides (mmol/L)	Baseline	1.05 ± 0.46	1.87 ± 1.19	2.184	NS
	Third month	0.96 ± 0.46 (adjusted: 0.98 ± 0.23)	1.26 ± 0.87 (adjusted: 1.25 ± 0.20)	0.777	NS
	*F*	0.207	1.317	-	-
	*p*	NS	NS	-	-

*F*-value and *p*-value for comparisons by one-way analysis of variance or covariance analysis for between-group differences at the third month, with adjusted data presented as mean ± standard error.BMI—body mass index; LDL-C—low density lipoprotein cholesterol; HDL-C—high density lipoprotein cholesterol; NS: differences were not significant.

**Table 7:** Comparison of antidiabetic drugs between the two groups.

Study Period		Peanut (*n* = 11)	Almond (*n* = 14)	*F*/*χ*^2^	*p*
		$\bar{x}$ ± SD/*n* (%)	$\bar{x}$ ± SD/*n* (%)		
Baseline	No	1 (9.1%)	2 (14.3%)	0.423 ^a^	NS
	Oral antidiabetic drugs	7 (63.6%)	9 (64.3%)		
	Insulin	0 (0%)	0 (0%)		
	Both	3 (27.3)	3 (21.4%)		
Third month	No	1 (9.1%)	1 (7.1%)	0.581 ^a^	NS
	Oral antidiabetic drugs	7 (63.6%)	10 (71.4%)		
	Insulin	0 (0%)	0 (0%)		
	Both	3 (27.3)	3 (21.4%)		
Insulin dose (IU)	Baseline	28.33 ± 11.59	36.00 ± 24.58	0.239 ^b^	NS
	Third month	27.00 ± 10.82 (adjusted: 30.19 ± 1.18 ^d^)	33.33 ± 20.03 (adjusted: 30.14 ± 1.18 ^d^)	0.001 ^c^	NS

^a^ Fisher’s exact test; ^b^ one-way analysis of variance; ^c^ covariance analysis; ^d^ standard error; NS: differences were not significant.

**Table 8:** Comparison of interleukin-6 (IL-6) ($\bar{x}$ ± s, pg/mL) in the two groups.

Study Period	Peanut (*n* = 11)	Almond (*n* = 14)	*F*	*p*
Baseline	12.78 ± 30.62	2.18 ± 1.10	1.696	NS
Third month	10.65 ± 26.91 (adjusted: 5.44 ± 0.52)	2.70 ± 1.83 (adjusted: 6.79 ± 0.45)	3.761	NS
*F*	0.030	0.832	-	-
*p*	NS	NS	-	-

*p*-value for comparisons by one-way analysis of variance or covariance analysis for between-group differences at the third month, with adjusted data presented as mean ± standard error. NS: differences were not significant.

**Table 9:** Comparison of albumin/creatinine (ACR) ($\bar{x}$ ± s, mg/g) in the two groups.

Study Period	Peanut (*n* = 11)	Almond (*n* = 14)	*F*	*p*
Baseline	25.58 ± 26.40	18.53 ± 16.19	0.679	NS
Third month	31.47 ± 48.70 (adjusted: 25.55 ± 5.26)	17.88 ± 21.87 (adjusted: 22.54 ± 4.65)	0.182	NS
*F*	0.124	0.008	-	-
*p*	NS	NS	-	-

*p*-value for comparisons by one-way analysis of variance or covariance analysis for between-group difference at the third month, with adjusted data presented as mean ± standard error. ACR—ratio of urinary albumin/creatinine; NS: differences were not significant.
